# In Memoriam: Frank John Fenner (1914–2010)

**DOI:** 10.3201/eid1704.IM1704

**Published:** 2011-04

**Authors:** Frederick A. Murphy

**Affiliations:** Author affiliation: University of Texas Medical Branch, Galveston, Texas, USA

**Keywords:** Frank John Fenner, virology, in memoriam, obituary

Frank John Fenner (Figure), one of the world’s most distinguished virologists and a dear friend of many colleagues around the world, died in Canberra, Australia, on November 22, 2010, at the age of 95. This In Memoriam must be different from those usually published here. After all, quite detailed pieces are anticipated from the Australian Academy of Sciences, the Royal Society, et al., and Frank had published a comprehensive autobiography (*1*). Thus, there is opportunity to present personal memories, hoping to provide more of a sense of the man, the colleague, and the friend of so many members of the global virology community. This tribute seems to fit in with the closing paragraphs of Frank’s autobiography, in which he reflects on friendship and special friends.

**Figure:**
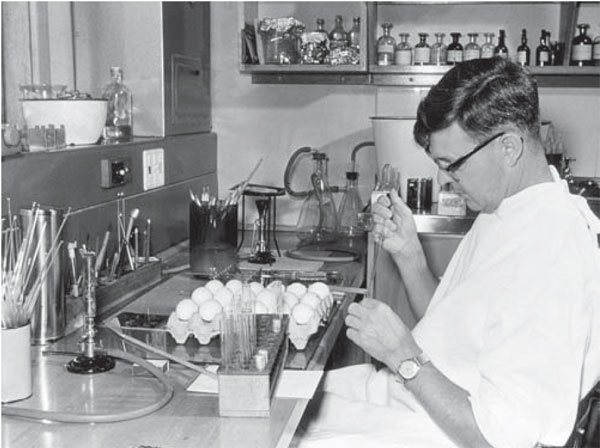
Frank Fenner at the John Curtin School of Medical Research, Canberra, Australia, inoculating embryonating eggs with myxoma virus, 1950. Used with permission of the John Curtin School of Medical Research.

Frank was born in Ballarat, Victoria, Australia, but his family moved to Adelaide, South Australia, when he was 2 years old. He received a bachelor of medicine and surgery degree (1938) and a doctor of medicine degree (1942) at the University of Adelaide. During 1940–1946, he was an officer (Captain, Major) in the Australian Army Medical Corps and served in Australia, Palestine, Egypt, New Guinea, and Borneo. At various times, he worked as a medical officer in a field ambulance and casualty clearing station, a pathologist in a general hospital, and, most prominently, as a malariologist. For his work in combating malaria in Papua New Guinea, he was made Member of The Order of the British Empire. As Frank noted later, the highlight of his military service was meeting and marrying a nurse, his wonderful wife, Ellen, known to all as Bobbie. Bobbie died in 1995, but ever after Frank said that his marriage was the most important part of his life. Frank is survived by his daughter, Marilyn, grandson, Simon, granddaughter Sally, and great-grandson Jasper.

After his war-time service, Frank was recruited to work at The Walter and Eliza Hall Institute of Medical Research in Melbourne by Sir Frank Macfarlane Burnet. Initially, they worked on ectromelia virus infection in mice (to explain his work, Frank coined the term mousepox). In 1949, he received a fellowship to study at the Rockefeller Institute for Medical Research in New York City, where he worked in the laboratory of René Dubos. While Dubos worked on *Mycobacterium tuberculosis*, Frank worked on *M*. *ulcerans*, the etiologic agent of Buruli ulcer, the third most common mycobacterial disease worldwide after tuberculosis and leprosy.

Returning to Australia in 1949, Frank was appointed Professor of Microbiology at the new John Curtin School of Medical Research (JCSMR), a unit of the Australian National University (ANU), in Canberra. He then began studying myxoma virus infection in rabbits. Throughout the 1940s and 1950s, Australia had severe rabbit plagues. Frank’s classic work on myxomatosis culminated in his classic 1948 paper (*2*). This paper includes a description of the progression of mousepox infection in mice, from the time/site of virus entry, to the time/sites of infection of major target organs, to the time/sites essential for virus transmission. It is still featured in all virology texts, and it marks the beginning of modern research in viral pathogenesis. At the same time, this work provided the scientific basis for release of myxoma virus for biologic control of rabbits. This work became the foundation for understanding the parallel evolution of the virus toward lesser virulence and the evolution of the rabbit toward more resistance to infection; it is another classic concept featured in virology texts.

Frank became director of the JCSMR in 1967 and served in this role until 1973. He made major changes in the school across broad areas of biomedical science, developing and changing the departments of genetics, medical chemistry, clinical science, human biology, immunology, and pharmacology, and guided the microbiology, immunology, and virology faculties toward world prominence. Imagine one building housing (not all at the same time) such greats as Gordon Ada, Alan Bellett, Bob Blanden, Stephen Boyden, John Cairns, Peter Cooper, Colin Courtice, Peter Doherty, Jack Eccles, Stephen Fazekas de St. Groth, Adrian Gibbs, Alfred Gottschalk, Royal Hawkes, Dick Johnson, Bill Joklik, Kevin Lafferty, Graeme Laver, Fritz Lehmann-Grube, George Mackaness, Barrie Marmion, Ian Marshall, Brian McAuslan, Peter McCullagh, Cedric Mims, Bede Morris, Chris Parrish, Ian Ramshaw, Rob Webster, David White, and Gwen Woodroofe.

And on the side, Frank published 23 books, of which 4 have become symbols of the march of virology through one of its most expansive eras: The Biology of Animal Viruses (2 editions) (*3*), written with Brian McAuslan, Joe Sambrook, David White, and Cedric Mims; Myxomatosis (recently republished) (*4*), written with Francis Ratcliffe; Medical Virology (4 editions) (*5*), written with David White; and Veterinary Virology (3 editions) (*6*), written with Paul Gibbs, Michael Studdert, Peter Bachmann, David White, Rudi Rott, Marian Horzinek, and Fred Murphy. All of Frank’s books are incredibly readable; he cited the influence of his father in developing his writing style: “Always generalize.” Somehow, in books so full of hard experimental data and proven facts, he always seamlessly added his magic, his interpretative skills, and his clear use of the King’s English.

In 1977, the World Health Organization (WHO) named Frank the chairman of the Global Commission for the Certification of Smallpox Eradication. The last known case of naturally transmitted smallpox occurred in Somalia in 1977, and WHO set in place a comprehensive global program to make sure that pockets of infection had not been overlooked. Eradication of the disease has been regarded as one of the greatest achievements in human history, and it was Frank’s great honor to officially pronounce global eradication to the World Health Assembly in May 1980. He later said that making this pronouncement stood out most among his many achievements: “[The pronouncement] was accepted unanimously by the World Health Assembly on that day.”

Frank had an abiding interest in the environment, and in 1973 was appointed foundation director of the Centre for Resources and Environmental Studies at ANU; he held this position until his retirement in 1979. The Centre became part of the Fenner School of Environment and Society in 2007. It was in this role, late in his career, that Frank publicly expressed a gloomy prospect for the future of humankind: environmental degradation, global warming, overpopulation. In retrospect, this view now seems at odds with Frank’s otherwise positive view of life, especially in regard to the grand prospects of advances in medical science in support of human welfare.

Frank played many national and international leadership roles (*1*), but 2 in particular were of great interest to him and central in the advance of science in Australia and virology internationally. The first role was in the Australian Academy of Sciences. Frank was in the first group of scientists to become fellows of the Australian Academy of Sciences, and over the years served in several roles. He served as secretary for biologic sciences, where he spearheaded studies of fauna in Australia and became a leader and financial supporter of environmental conservation programs. Later, this grew into the Fenner Conferences on the Environment and the Fenner Medal for Plant and Animal Sciences.

The second role was in the International Committee on the Taxonomy of Viruses. Frank had been interested in virus taxonomy from his first days working in the field. He became a charter member of the International Committee on Nomenclature of Viruses (changed to the International Committee on the Taxonomy of Viruses in 1973) as it was established by Sir Christopher Andrewes, André Lwoff, and Peter Wildy at the International Congress of Microbiology in Moscow in 1966. Frank was unable to attend the next Congress in Mexico City in 1970, but in his absence was elected the second president of the committee (the first president was Peter Wildy). These were formative days; the basic structure of virus taxonomy as we know it today emerged from the work of the committee, under the presidencies of Peter Wildy and Frank Fenner, as the committee met at the first 3 International Congresses of Virology (Helsinki, 1968; Budapest, 1971; and Madrid, 1974) (*7*).

Over the years Frank received many honors (*1*). However, some are listed to illustrate his preeminent place in the world of science, virology, and public service: Companion of the Order of St. Michael and St. George, Companion of the Order of Australia, Foreign Associate of the US National Academy of Sciences, the Australian Prime Minister’s Prize for Science, the Japan Prize, the Copley Medal of the Royal Society, the WHO Medal, and the Albert Einstein World Award for Science. Frank was also proud that the Frank Fenner Building, which houses the ANU Medical School and Faculty of Science, and a residential college, Fenner Hall, are named in his honor.

Turning to a more personal viewpoint, I was fortunate to spend 1970–1971 at the JCSMR as an honorary fellow in the laboratory of Cedric Mims. At the time, Frank was director of the School. It was an amazing time, with the outstanding virology faculty well in place, most having been recruited by Frank in preceding years. It was an institution where conceptualization preceded experimentation, that is, where everyone thought a lot about the anticipated results and meaning of his or her experimental work beforehand.

One of the keys to the intellectual energy level was morning coffee, a lost art. At ≈10:30 am, small groups formed in the departmental lounge, and discussion began, often leading to cryptic notes written on napkins, in some instances leading to collaborative papers in the Journal of Experimental Medicine, the Journal of Infectious Diseases, or such. On occasion, small meetings were held in Frank’s office, in some instances with guests, with collaborative projects involving virologists from other institutions and other countries following. I recall one such meeting in Frank’s office where the director of the Commonwealth Scientific and Industrial Research Organisation had dropped by to bring up a new wrinkle in the use of myxoma virus for rabbit control across Australia. In all this discussion, there was an infectious verve, a sense that the edges of the virologic and immunologic sciences were about to be breached, again. Upon returning to the Centers for Disease Control, I tried to transplant this scientific lifestyle, and although successful within our small unit, I realized that by achieving such a productive and satisfying lifestyle across the entirety of the JCSMR, Frank had again used his magic.

Later, another of Frank’s characteristics was made clear to me: his prodigious capacity for work and for the highest ethical standard in dealing with data, his colleagues, and his students. He wrote (*1*), “From childhood, I have been an early riser… getting up when I woke at about 5 am… usually arriving at the School between 6 and 7 am.” I recall that Frank’s car was always in the first parking space; if it was not, it meant he was out of town. This work ethic extended to the writing/editing of various editions of the books Veterinary Virology (*6*) and Medical Virology (*5*). Frank would come to Atlanta for a week at a time and stay at our house. With thousands of marginal notes made beforehand, we would spend endless hours going over chapters. Usually I thought the effect was chaotic, but then a few weeks later I would be sent the most lucid, organized drafts, all in Frank’s uniquely smooth, clear, expansive style. I recall times when my wife would call that dinner was ready and I would crumple in anticipation of a drink and a bit of a rest, only to be rejoined after a wonderful dinner and family conversation by Frank’s gentle voice, “time to get back to work.” I recall that in all this writing/editing, Frank held to a high level of objectivity and honesty in the prose; this was rather like the concept of “evidence-based medicine” of today. I attribute this skill to Frank’s sense that the written word must reflect an underlying ethical standard, one that we all should emulate.

I could go on, but perhaps there is place for one more personal memory (I hope my memories prompt others to recall their own). This memory can start with Frank’s vignette in his autobiography (*1*): “Bozeman and Yellowstone National Park, 11–25 July 1997: I had been invited to give the Edwin H. Lennette Memorial Lecture at the annual meeting of the American Society for Virology, in Bozeman, Montana, in July 1997. Fred Murphy got in touch with me well before the meeting and suggested that I accompany his family on a week’s trip through Yellowstone and Grand Teton National Parks. Fred had an RV (recreational vehicle), which had beds, shower, toilet, stove, and refrigerator. Fred, Irene, son Rick and his 2 young boys, son Tim and his wife, son Terence and his wife, and I travelled from Bozeman and throughout the parks in the RV; his sons had also brought their bicycles. We had a wonderful trip all around Yellowstone, which was not only the first national park in the USA, but one of the most wonderful in the world, with entrancing hot springs, geysers and waterfalls. We also had a great float trip down the Snake River beneath the Tetons. Then we went to Bozeman, where the meeting was very interesting. I gave my lecture and saw a lot of old friends, including Joe Esposito, Grant McFadden, Olin Kew, Dick Moyer and Mary Estes.”

This trip was one of the most memorable camping trips the Murphy family had ever taken, and Frank became family immediately. I recall seeing Frank walking down a wide trail with 2 of my daughters-in-law, 1 on each arm, all chatting and laughing; I have always wondered what they were talking about. I recall finding a bottle of scotch in a cupboard of the RV, which Frank and I used pharmaceutically at the end of each day of adventure as we sat before dinner in the Murphy perfectly matched lawn chairs (from yard sales; no 2 alike). The bottle eventually was emptied and we lamented its passing, but the next evening as I perchance opened the cupboard, there was a new bottle. Frank had mysteriously found a place to replenish the elixir, but of course he never explained his action. I must admit that as we sat and sipped that scotch the topic of conversation was often virology. Afterward, my son Rick sent Frank a copy of his video of the trip, so there was much email traffic back-and-forth reliving each adventure. Life at its fullest.

Does all this capture Frank Fenner, the man, the friend who has died? If not, then words cannot serve what memories can. Whenever we think of the rise of virology from its roots in pathology and the infectious disease sciences, to its flowering in the first laboratory experiments in viral biology and pathogenesis, and to the beginnings of molecular virology, the name Frank Fenner will be remembered. Whenever we who knew him as a friend think of the emergence of virology as a distinct discipline, at the time of the first International Congresses of Virology (1968 forward), his role will be remembered with great fondness. Whenever we extend these memories to the more personal aspects of life, Frank’s quiet, unassuming character, a character with a steel spine and great insight and understanding of people, will be recalled, again with fondness mixed with great respect. He will never be forgotten.
